# The Effect of Q-Switched Nd:YAG 1064 nm/532 nm Laser in the Treatment of Onychomycosis In Vivo

**DOI:** 10.1155/2013/379725

**Published:** 2013-12-14

**Authors:** Kostas Kalokasidis, Meltem Onder, Myrto-Georgia Trakatelli, Bertrand Richert, Klaus Fritz

**Affiliations:** ^1^Dermatology and Laser Clinic, 88 Tsimiski Street, 54622 Thessaloniki, Greece; ^2^Dermatology and Laser Center, Reduitstrare 13, 76829 Landau, Germany; ^3^Gazi University Medical Faculty, Department of Dermatology, 06510 Ankara, Turkey; ^4^Aristotle University School of Medicine, Second Department of Dermatology and Venereology, 54622 Thessaloniki, Greece; ^5^Université Libre de Bruxelles, Department CHU Brugmann-Saint Pierre, 1050 Brussels, Belgium; ^6^Carol Davila University of Medicine, Dionisie Lupu Street, 020021 Bucharest, Romania; ^7^Osnabrueck University, Sedanstraße 115, 49090 Osnabrueck, Germany; ^8^Bern University, Department of Dermatology, 117 Inselspital, 3010 Bern, Switzerland

## Abstract

In this prospective clinical study, the Q-Switched Nd:YAG 1064 nm/532 nm laser (Light Age, Inc., Somerset, NJ, USA) was used on 131 onychomycosis subjects (94 females, 37 males; ages 18 to 68 years). Mycotic cultures were taken and fungus types were detected. The laser protocol included two sessions with a one-month interval. Treatment duration was approximately 15 minutes per session and patients were observed over a 3-month time period. Laser fluencies of 14 J/cm^2^ were applied at 9 billionths of a second pulse duration and at 5 Hz frequency. Follow-up was performed at 3 months with mycological cultures. Before and after digital photographs were taken. Adverse effects were recorded and all participants completed “self-evaluation questionnaires” rating their level of satisfaction. All subjects were well satisfied with the treatments, there were no noticeable side effects, and no significant differences were found treating men versus women. At the 3-month follow-up 95.42% of the patients were laboratory mycologically cured of fungal infection. This clinical study demonstrates that fungal nail infections can be effectively and safely treated with Q-Switched Nd:YAG 1064 nm/532 nm laser. It can also be combined with systemic oral antifungals providing more limited treatment time.

## 1. Introduction

Onychomycosis is defined as a fungal infection of the nail that expands slowly and if left untreated leads to complete destruction of the nail plate. Onychomycosis can be dermatophytic (99%) and/or nondermatophytic (1%) (including yeasts) infections of the nail plate.

The dermatophytes *Trichophyton rubrum* and *Trichophyton mentagrophytes* are the most common causative pathogens responsible for up to 90% of all cases [1]. Onychomycosis represents about 30% of all dermatophyte infections and accounts for 18%–40% of all nail disorders. The prevalence of onychomycosis ranges between 2% and 28% of the general population and it is estimated to be significantly higher in specific populations such as in diabetes mellitus, the immunosuppressed, and elderly [2, 3].

Among the nondermatophytes, the yeast *Candida albicans*, *Candida tropicalis*, *aspergillus*, and other molds may be responsible. It usually represents contamination and is an emerging problem in HIV patients.

Toenails are far more likely to be involved than fingernails. Initially solitary nails are involved; later, many may be infected, but often one or more can stay disease-free. Onychomycosis has no tendency for spontaneous remission and should be considered as a problem with serious medical, social, and emotional extensions, not solely a cosmetic problem. The primary concerns of the patients are the risk of spread to other nails or to people in their environment. Others consider their deformed nails as unattractive to other people, which may lead to lower self-esteem, a sense of inadequacy, and even depression [4, 5]. In addition to these social and emotional problems, onychomycosis is a serious medical problem that can be the source of further fungal infections to surrounding tissues. Also, it may predispose patients to secondary bacterial infections leading to localized paronychia and perhaps worse and deeper infections such as erysipelas-cellulitis, especially in the high-risk groups such as diabetics [6, 7]. Clinically it can cause varying degrees of pain or discomfort (especially in walking) and problems in cutting nails.

Classical treatment options include mechanical and chemical debridement, topical antifungal lacquers, systemic antifungal drugs, and finally various combinations of the above. The most effective mono-therapies for onychomycosis are antifungal agents which have been the gold standard and mainstay of therapy for years. The downside of the antifungals are that they require blood testing to monitor the liver because they are systemic and also that they require long treatment courses (approximately 6 months for toenails and 4 months for fingernails). This requires liver function-transaminases and kidney function blot test control. Patients may also receive concomitant medications for comorbidities, so there is also the issue of drug interactions. Additionally, long lasting treatment means high treatment costs for both the patient and health insurers. Finally, high recurrence rates have been described, 22% three years after completion of treatment and higher recurrence rates at five years follow-up [8–10].

Recently, lasers have emerged as potential new treatment modalities. These treatments offer the advantage of having few contraindications and minimal side effects [11–13]. Laser energy has the potential to eliminate microorganisms. Vural et al. recently demonstrated direct inhibitory activity of laser energy on *T. rubrum* isolates in vitro [14]. Manevitch et al. recently published the direct antifungal effect of the femtosecond laser on *T. rubrum* onychomycosis as well [7]. The laser must have the ability to penetrate under the nail plate in order to reach the fungi colonies of the nail bed and nail matrix. When it gets to that point it should selectively deliver laser energy to fungi while respecting the surrounding healthy tissues.

In this study we planned to evaluate the effect of the neodymium: yttrium-aluminum-garnet (Nd:YAG) 1064 nm/532 nm laser in the treatment of onychomycosis in vivo.

## 2. Material and Methods

### 2.1. Nail Sampling and Fungal Cultures

Nail cuttings sized 2 × 3 mm were obtained from patients with clinical suspicion of onychomycosis. After direct microscopy to observe spores, hyphae, mycelia, and colonies of the latter, samples were plated on *Sabouraud glucose agars* with *cyclohexamide* to select for dermatophytes, in order to verify fungal infection. Cultures were incubated at 28°C for 3 weeks until fungal colonies developed.

### 2.2. Evaluation of Fungal Elimination

Before the treatment culture was performed and 4 weeks after the second treatment session (8 weeks after the first positive culture), culture was repeated. Mycological cure is defined as negative microscopy and culture. Clinical cure is associated with the alteration of the percentages of disease-free nails. Complete cure is accepted as the combination of mycological and clinical cure. Three months after the first treatment session, laser treatment was evaluated [15, 16].

### 2.3. Inclusion Criteria

To take part in the study each patient had to have one or more toenail and/or fingernail fungal infections of the following types: distal subungual onychomycosis, proximal subungual onychomycosis, superficial white onychomycosis, or total dystrophic type onychomycosis. Patients with diabetes mellitus, immunocompromised patients, and organ transplant patients were also included, although we considered that these patient groups success rates could be considerably less.

### 2.4. Exclusion Criteria

Patients who used systemic antifungal or isoretinoin within 6 months of the first scheduled laser session were excluded. The following conditions, which can cause various physiological changes to the nail plate, were also excluded: subungual hematoma, nevoid subungual formation, bacterial nail infections, concomitant nail disorders due to psoriasis, atopic dermatitis, lichen planus, and pregnant women were not included.

### 2.5. Pretreatment

As onychomycosis causes significant thickening (hyperkeratosis) of the nail plate, before starting our laser sessions we performed the mechanical debridement of any excessive nail thickness. This procedure was conducted with a file by a trained podiatrist. This mechanical debridement alone does not constitute an effective treatment, but it helps the laser penetrate under the nail plate to reach the fungal colonies of the nail bed and nail matrix.

### 2.6. Grading the Severity of Onychomycosis: Onychomycosis Severity Index

The Onychomycosis Severity Index (OSI) score is obtained by multiplying the score for the area of involvement with a range of 0–5 (1–10% is rated with 1, 11–25% with 2, 26–50% with 3, 51–75% with 4, and finally 76–100% with 5) by the score for the proximity of disease to the matrix with also a range of 1–5. Ten points are added for the presence of a longitudinal streak or a patch (dermatophytoma) or for greater than 2 mm of subungual hyperkeratosis. Mild onychomycosis corresponds to a score of 1 through 5; moderate, 6 through 15; and severe, 16 through 35. All patients were examined monthly for the evidence of proximal extension of the nail bed lesion. Any proximal extension of the lesion during treatment was a treatment failure [17, 18].

### 2.7. Laser Irradiation

The irradiation was performed with a Q-Switched Nd:YAG 1064 nm (Q-Clear, Light Age, Somerset, New Jersey, USA). Laser protocol was performed with 2.5 mm spot size and a power level of 4 which delivers 14 joules/cm^2^, 9 billionths of a second pulse duration, and a 5 Hz frequency.

The second pass was done with the same laser operating at 532 nm Nd:YAG with the following parameters: 2.5 mm spot size and a power level of 4 which delivers 14 joules/cm^2^, 9 billionths of a second pulse duration, and a 5 Hz frequency. No local anesthesia was applied preoperatively.

In one session two passes across each nail plate were performed with two minutes pauses between each pass. The first pass was performed with the 1064 nm Nd:YAG laser. Each nail was fully covered with a laser beam, including the areas of the hyponychium and the proximal and lateral nail folds. After a two minute intermission the second pass was performed with the 532 nm Nd:YAG, fully covering the nail plate but not the hyponychium and nail folds. All patients were also evaluated with posttreatment fungal cultures.

Postoperative analgesic treatment was not required. No prophylactic antibiotics or antivirals were given to any patient.

The full treatment consisted of two sessions executed on days 0 and 30. Nails were photographed with a high-resolution digital camera before treatment at day 0 (pre-treatment photograph). Follow-up visits were done at day 30 (before the second session). Photographs were taken again using the same camera settings, with lighting and nail position at baseline and day 60.

## 3. Results

### 3.1. Clinical Onychomycosis Types

Patients had all four major clinical types of onychomycosis: distal subungual onychomycosis, proximal subungual onychomycosis, superficial white onychomycosis, or dystrophic type onychomycosis. Another group is onychomycosis that affects only the lateral edge. The clinical onychomycosis types separated by gender and age group are given in Table 1.

Distal subungual is the most common clinical type of onychomycosis among both genders and all age groups since it appears in 123 (93.9%) of the total patients, followed by lateral edge (in 47 patients (35.9%)), dystrophic type (in 13 or 9.9%), superficial white (in 2 patients or 1.5%), and, last, proximal subungual (in only 1 patient or 0.8%). Moreover, 94.7% of female patients, 91.9% of the males, 95% of patients under 30 years old, 93% between 30 and 60 years old, and 95.8% over 60 suffer from distal subungual. The corresponding counts and percents for the rest of the clinical types of onychomycosis may be seen in Table 1.

### 3.2. Fungus Types

The most frequent fungus found among treated patients was *T. rubrum* (in 108 patients or 82.3%), followed by *Candida* (in 19 patients 14.6%) and then *Trichophyton mentagrophytes* (in 4 patients 3.1%).  Table 2 presents the types of fungi found in patient populations and their percentages. The fungus types can also be seen by patient's ages and genders.

### 3.3. Severity of Onychomycosis

Table 3 shows all patients according to onychomycosis severity.

Regarding the severity of onychomycosis, severe onychomycosis seems to be more frequent in men (78.4% versus 62.8%). A chi-square test for the differences between genders suggested that those differences are not statistically significant at any significance level (*χ* ^2^ = 3.681, *P* = 0.159). We draw the same conclusions from a chi-square test for the differences in age (*χ* ^2^ = 3.002, *P* = 0.557).

We also evaluated our patients according to great nail and/or multiple nail involvement (Table 4).

### 3.4. Mycologic Cure of Nail Fungal Infections

At 3-month follow-up 125 patients (95.42%) showed mycological cure (negative microscopy and culture). There was no treatment failure (proximal extension of the lesion during treatment). Clinical cure is associated with the alteration of percentages of disease-free nail. We find a change of >76% as excellent response, 51–75% as very good response, 26–50 as good response, 6–25% as moderate response, and >5% as low response to treatment.

It can be seen in Table 5 that the clinical type of onychomycosis seems to have an important influence on response: “distal subungual” had the best response followed by “lateral edge, dystrophic type, and superficial white”; however “proximal subungual” type showed the lowest response.

Dermatophytes (*T. rubrum*) seem to have the best response rate followed by *Trichophyton mentagrophytes* and *Candida* comes last. Paradoxically, *moderate *onychomycosis showed the best results, while *mild* is next and *severe* last.

**Figure 1:**
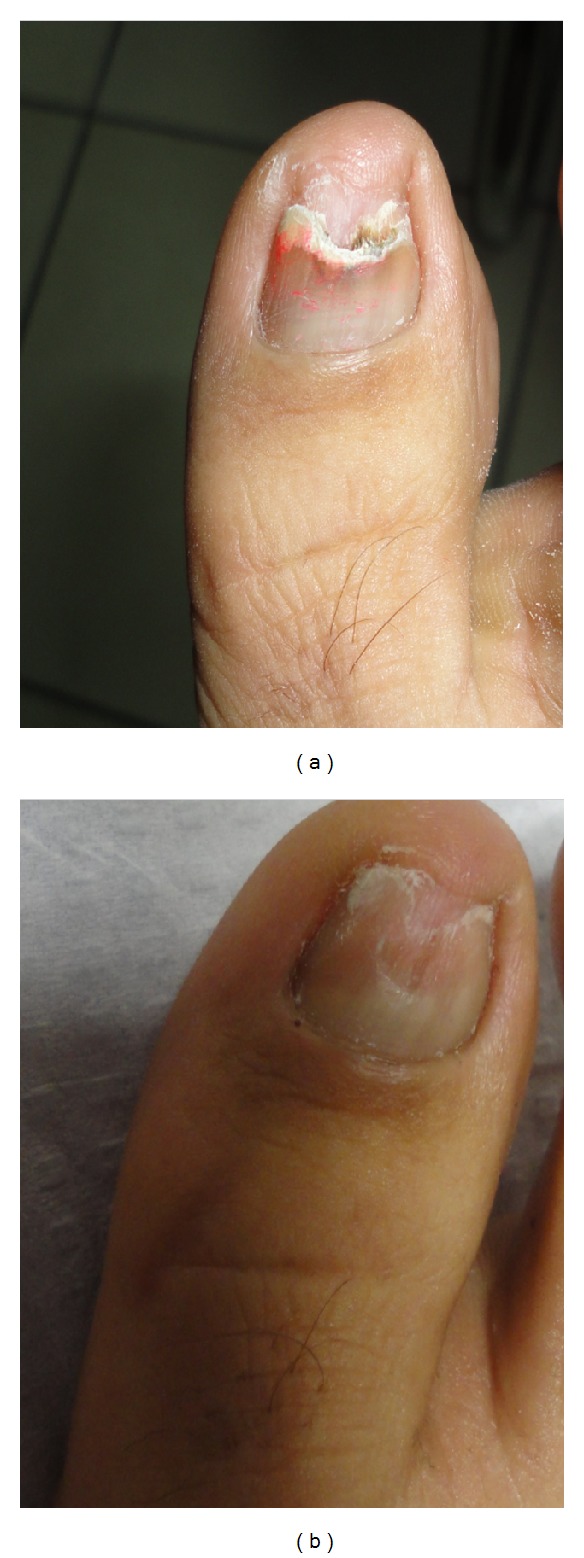
(a) 68-year-old female patient, before treatment. *Trichophyton rubrum* was isolated on mycological testing. Onychomycosis severity index (OSI) was 16. (b) After treatment with good improvement. OSI is 6 showing 69.5% improvement.

**Figure 2:**
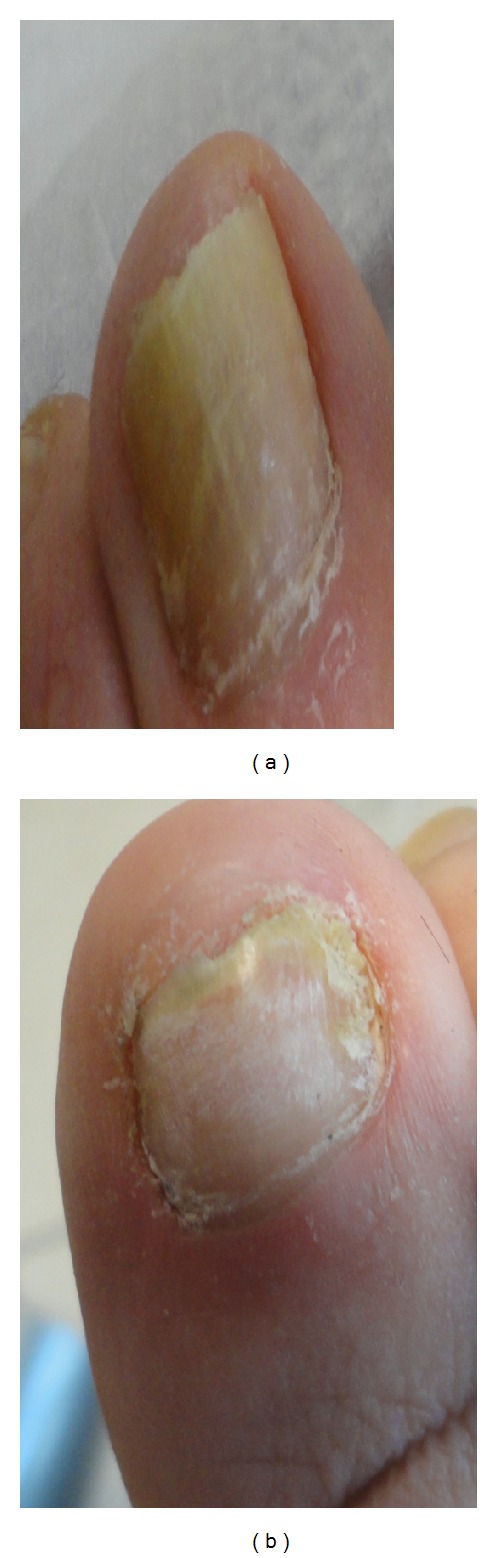
(a) 64-year-old female patient before treatment. *Trichophyton rubrum* was isolated on mycological testing. OSI is 26. (b) After treatment with great improvement. OSI is 9 showing 65.38% improvement.

**Figure 3:**
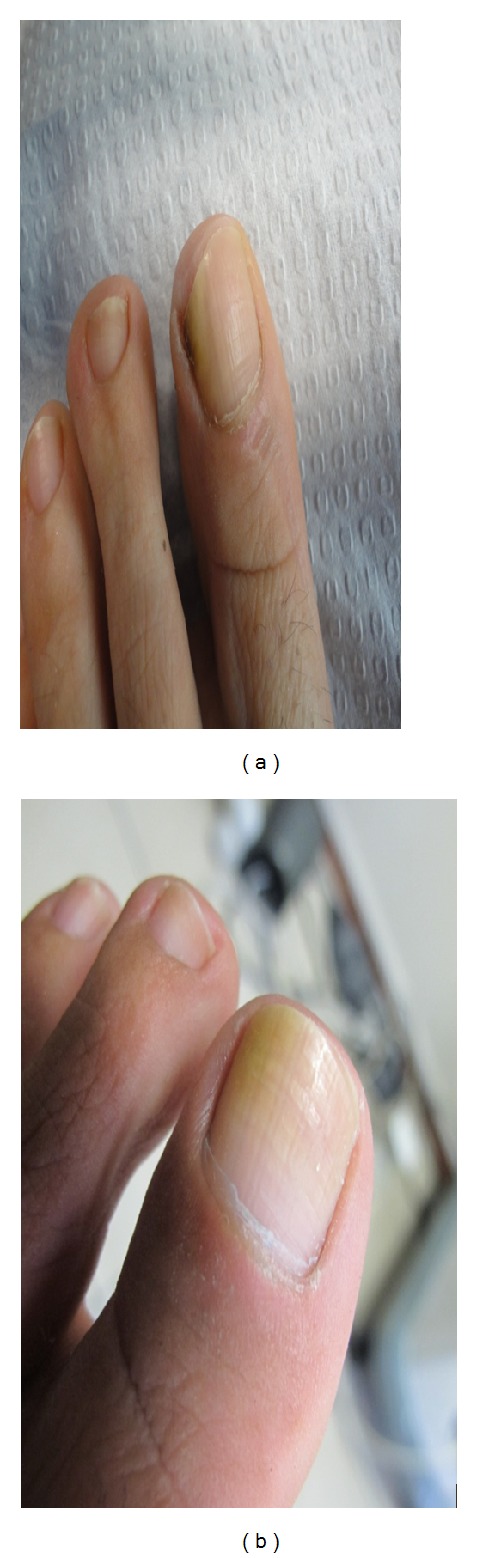
(a) 48-year-old female patient before treatment*. Trichophyton rubrum* was isolated on mycological testing. OSI was 9. (b) After treatment with great improvement. OSI is 2, showing 77.78% improvement.

**Figure 4:**
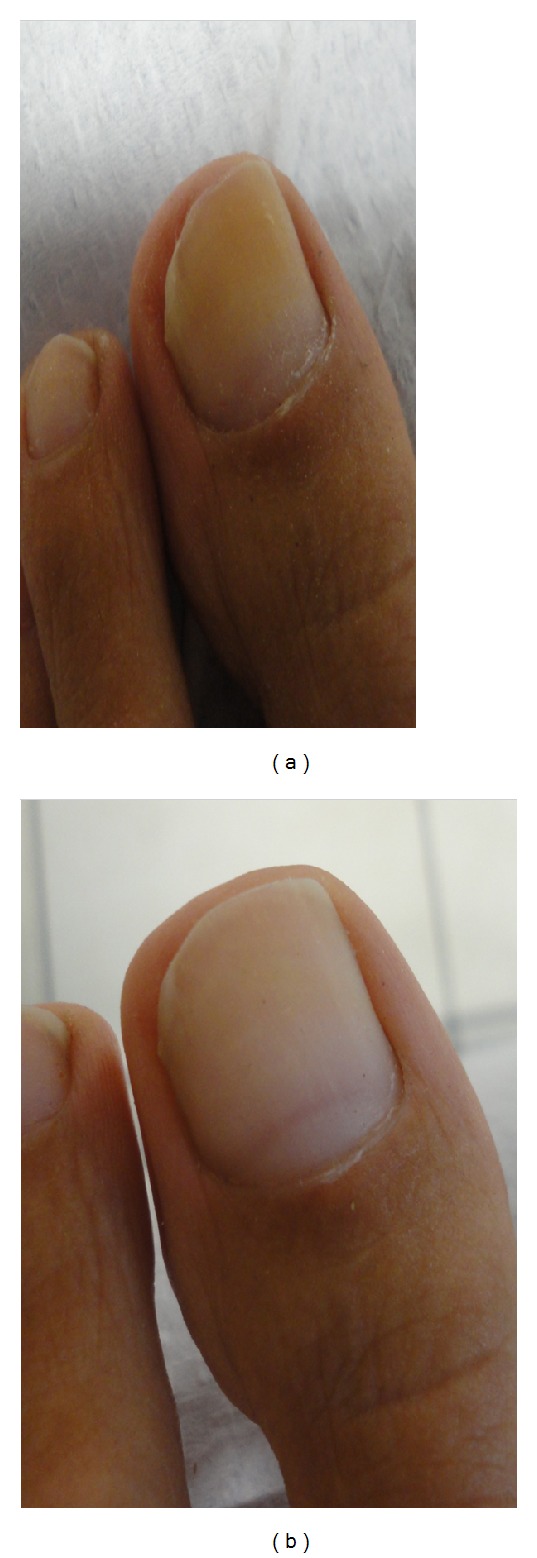
(a) 28-year-old female patient before treatment. *Trichophyton rubrum* was isolated on mycological testing. OSI was 12. (b) After treatment with great improvement. OSI is 1, showing 91.67% improvement.

**Figure 5:**
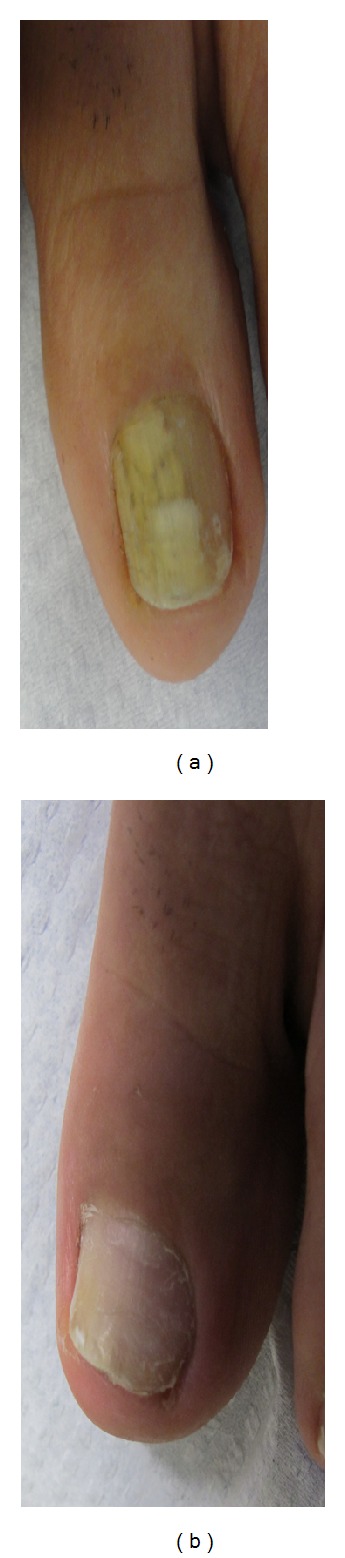
(a) 60-year-old female patient, before treatment. *Verticillium sp.* was isolated on mycological testing. OSI was 30. (b) After treatment with great improvement. OSI is 4 showing 86.67% improvement.

**Figure 6:**
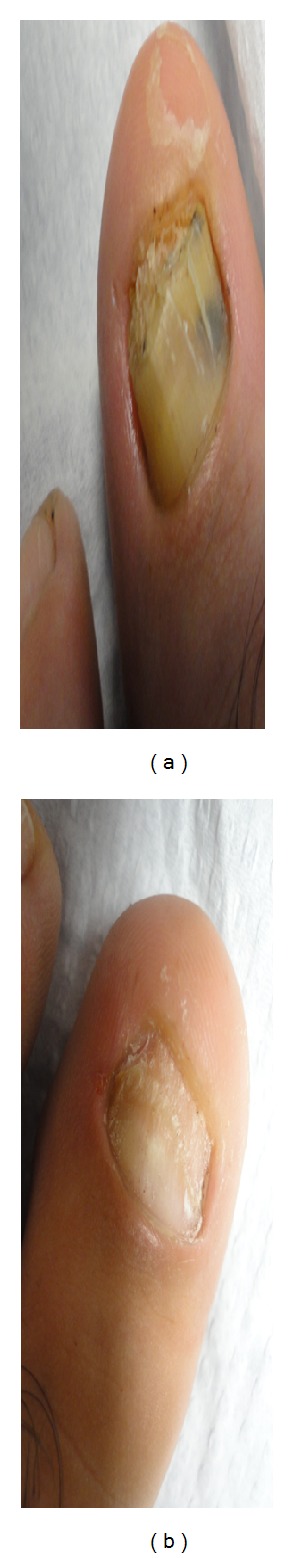
(a) 32-year-old male patient, before treatment. *Trichophyton rubrum* was isolated on mycological testing. OSI was 35. (b) After treatment with great improvement. OSI is 12 showing 65.71% improvement.

**Figure 7:**
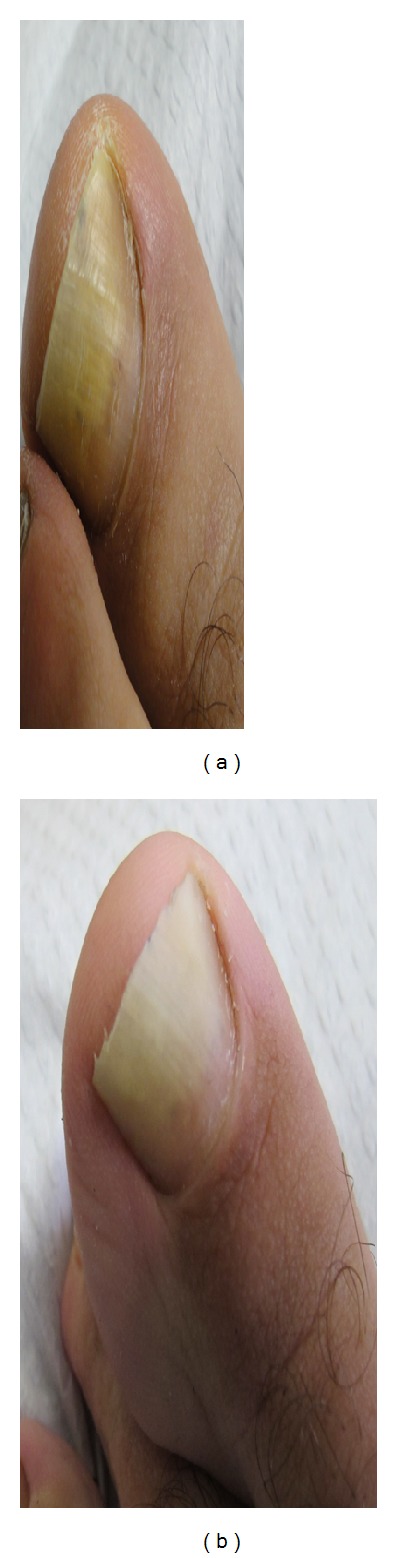
(a) 58-year-old male patient, before treatment. *Trichophyton rubrum* was isolated on mycological testing. OSI was 30. (b) After treatment with great improvement. OSI is 9 showing 70.00% improvement.

The age group under 30 revealed the best results, additionally women showed the best response (Figures 1(a), 1(b), 2(a), 2(b), 3(a), 3(b), 4(a), 4(b), 5(a), 5(b), 6(a), 6(b), 7(a), and 7(b)).

Among the above differences, only three are statistically significant.

The following are the differences.

- Genders: women seem to be cured more effectively than men do at a 5% significance level (*f* = 5.237 and *P*-value = 0.024).
- Severity of onychomycosis: mild severity patients are cured most effectively, followed by moderate severity and lastly severe severity patients at a 1% significance level (*f* = 9.963 and *P*-value = 0.00).
- The responsible nail fungi:* T. rubrum* recedes more quickly after the cure, followed by* trichophyton mentagrophytes* and *Candida* at a 1% significance level (*f* = 15.347 and *P*-value = 0.00).

### 3.5. Adverse Event Evaluation

Most patients, 94 (83.21%), reported mild pain; 22 patients (16.79%) reported no pain. This “pain” sensation was described as “a stinging” during the 1064 nm pass and as “burning” during the 532 nm pass. None of the patients treated had severe or intolerable pain. No postoperative analgesic treatment was required. Interestingly many of patients developed a kind of pain resistance during the therapy, meaning they reported the highest level of pain during the first session. We believe this suggests the patients knew what to expect or that the fear of an unknown treatment no longer existed.

Patients were also asked to report all possible adverse events that could be related to our treatment. There were no reports of any other side effects.

## 4. Discussion

Treatment of onychomycosis is difficult. Laser treatment is considered by some authors to be a promising new method. Our study population comprised of 131 individuals. 15.3% of the participants in the study were below 30 years of age, 65.6% between 30 and 60 years, and finally 18.3%, were over 60 years old. These groups allowed us to maintain a large enough sample within each group to compare the effectiveness of the laser treatment on different age groups. Women were the 71.8% of our patient sample. This does not mean that onychomycosis occurs more frequently in women but that men may be more negligent in matters relating to the cosmetic appearance and hygiene of their feet.

In a recent paper Vural et al. showed that 1064 nm and 532 nm Q-Switched Nd:YAG laser systems had significant inhibitory effect upon *T. rubrum* isolates and caused colony growth inhibition in vitro [14]. It is well known that the efficacy of laser energy depends on the light-tissue interaction which is a function of wavelength, fluence, and tissue optics [7]. We have used various spot sizes in all power levels with our system. This can provide combinations which deliver different energy fluence. We found that the most powerful treatment was 14 joules/cm^2^; additionally, the 7.5 joules/cm^2^ (3.5 mm spot size and a power level of 4) was also effective. Since the treatment session is very well tolerated in the maximum energy fluence, we used these settings. We have noticed a significant improvement in the proximal portion of the nail where there was mild initial mycotic involvement. Our results were better especially in moderate severity patients. That seems reasonable as severe cases are accompanied by dermatophytoma or significant subungual hyperkeratosis, which require more time for the nail plate to restore. Poor prognostic indicators are the total dystrophic onychomycosis, the involvement of the lateral edge of the nail plate, and the involvement of the matrix [18–21]. The thick plate or subungual hyperkeratosis >2 mm histologically contains numerous air-filled spaces in which fungal spores can survive for weeks or months. These resting arthrospores do not form hyphae, so various antifungal agents have proven ineffective. This phenomenon, termed as dermatophytoma, can be seen as linear streaks or rounded white areas in the nail plate. The fungal elements are believed to be forming a biofilm, making them refractory to therapy [15, 22, 23]. Laser therapy seems not to be affected of this biofilm formation; this may explain why we achieved very good and good response in 67% of our severe cases. Moreover, old age, presence of immunosuppression, poor peripheral circulation and nonresponsive organisms (nondermatophyte molds), other dermatoses (e.g., nail psoriasis), and drug resistance are poor prognostic indicators [15, 23, 24]. With the laser we solve the problem of resistance. We suggest that we do not have nonresponsive cases but some poor responding fungi. As another example, occupational factors, as well as occlusive and prolonged contact with water, can contribute to poor response of treatment [21].

On the contrary, superficial white onychomycosis is associated with the best therapeutic response to antifungal drugs, and our results seem to agree with this [16, 19]. Our distal subungual clinical cases had good results as well. Even dystrophic types showed a very good and good response in 66% of the cases. This supports laser treatment efficacy. Laser treatment seems to outweigh classical treatments where involvement of the matrix, a thick plate, or subungual hyperkeratosis >2 mm are factors associated with poor outcome [15, 21].

The Q-Clear Laser System, in differentiation to other laser treatments, provides a selective, both thermal (photothermolytic) and mechanical (photomechanical), effect on the fungi. The mechanism of this fungal destruction may offer some differences. The inhibitory effect is likely due to more than simple nonspecific thermal damage. Denaturing one or more of the molecules within the pathogen may deactivate the fungi. Vural et al. discusses that 532 nm setting, which is well absorbed by red pigment in canthomegnin in *T. rubrum*, this wavelength generates mechanical damage in the irradiated fungal colony [14].

The 1064 nm setting is beyond the absorption spectrum of xanthomegnin, but its effectiveness is due to another absorbing chromophore, perhaps melanin, which is present in the fungal cell wall [14]. Melanin is an essential inhabitant of the fungal cell wall and has been described in many pathogenic species. The type of melanin varies, although it is commonly Dopa or pentaketide melanin. Moreover, the laser beam may initiate a photobiological or photochemical reaction that attacks the pathogen cell. We can also suggest a multiphoton dielectric breakdown at the fungal target as the cause of their destruction, while depth-selective thermal effects by the laser could also be occurring [7].

Another possible scenario is by inducing an immune response that attacks the organism. All of the above hypotheses explain how the surrounding host tissue cells are protected from this attack, with little or no collateral damage. The amount of energy delivered by our treatment session may serve as a deactivating dose. That amount of energy can deactivate 80–99% of the organisms present in an affected nail without instantly killing the fungal colonies but it can disable their ability to replicate or survive according to an apoptotic mechanism. Apoptosis, a physiological type of cell death, plays an important role in the selective deletion of cells in divergent situations of various tissues [25]. Induced apoptosis may cause direct DNA damage, for example, strand breaks, chromosomal aberrations, induction by transduced signals, for example, FAS/APO-1 transmembrane signals, and stress (heat) mediated death. Hyperthermia, a typical environmental stress, has long been known as toxic to cells. It has been recognized the mode of cell killing to be influenced by the severity of the heat treatment [26]. A number of reports have been published to demonstrate the induction of apoptosis by mild hyperthermia [27, 28].

We are waiting to assess our results following twelve months since the completion of treatment, which is the time required for complete regeneration of the nail plate. Additionally, we will follow the patients at greater time intervals to assess the occurrence of relapse. Zaias et al. recommended that the treatment of onychomycosis with oral antifungals should be continued until the mycotic nail bed had been completely replaced by a new mycotic bed (that requires about 12 months for toenails). With this treatment the authors achieved significantly better cure rates [18]. It may be that this maintenance therapy will provide a safety net for those at risk of relapse after the discontinuation of laser treatments.

In contrast to our results, recently Carney et al. evaluated thermal response and optical effects of a submillisecond neodymium: yttrium-aluminum-garnet (Nd:YAG) 1064 nm laser on common fungal nail pathogens and the clinical efficacy and safety laser of onychomycotic toenails. A fungicidal effect for *T. rubrum* was seen at 50°C after 15 minutes and for Epidermophyton floccosum at 50°C after 10 minutes. No inhibition was observed after laser treatment of fungal colonies or suspensions. In vivo treatment of toenails showed no improvement in Onychomycosis Severity Index score. They discussed that laser treatment of onychomycosis was not related to thermal damage or direct laser effects [29].

Similarly Hees et al. were also unable to show the effect of Nd:YAG laser on *T. rubrum *colonies. They assumed that the effect could be due to unspecific tissue heating with a subsequent increase in circulation and stimulation of immunologic process. They also discussed the associated risks of laser treatment with the use of higher densities [30]. Laser systems vary widely and it is understandable that there are differeing results. The Q-Clear's 1064/532 nm wavelengths and unique time-structured pulse profile specifically target the fungal elements, inducing a progressive and eventually lethal temperature increase. At the same time the low-absorption, high water content tissues (dermal), and vascular flow, allow rapid dissipation of absorbed energy, thus “antitargeting” the nail bed and other dermal tissues.

Competing “long pulse” systems presumably relay on bulk heating of fungal colonies in situ on the nail bed with the associated discomfort which necessitate multiple treatments and a high treatment failure rate. Some of the papers in the literature calling laser a failure were also only Petri dish studies which cannot replicate these in vitro applications.

Although some studies have yielded conflicting results, other studies like ours have shown some promise [31–34].

Zhang et al. had satisfactory results with the Nd:YAG without significant complications. They discussed that the thicker the nail plates the higher the laser energy needed to be. Different fungal strains may also have different sensitivities [32]. Hochman [33] and Bornstein et al. [34] described the formation of free radicals as well as the influence of the laser on cellular reaction. These results support our study.

Finally, we find the treatment of onychomycosis with this specific Q-Switched Nd:YAG, 1064 nm/532 nm laser in vivo as extremely promising and efficient. In addition, laser-based treatments have the advantage of a regimen that is devoid of mutagenic and genotoxic effects. They could be combined with systemic oral antifungals providing the benefit of limiting treatment time.

### 4.1. Weaknesses of the Research

Whereas the present study demonstrates the efficacy of the specific laser in the treatment of onychomycosis, we should keep in mind that negative cultures, that is, mycological cure, do not always constitute proof of clinical cure due to the well-known high rate of false-negative culture results.

## Figures and Tables

**Table 1:** Clinical onychomycosis types.

Patients	Total	Distal subungual	Proximal subungual	Superficial white	Dystrophic type	Lateral edge
	131 (100.0%)	123 (93.9%)	1 (0.8%)	2 (1.5%)	13 (9.9%)	47 (35.9%)
Gender						
Female	94 (71.8%)	89 (94.7%)	0 (0.0%)	2 (2.1%)	9 (9.6%)	26 (27.7%)
Male	37 (28.2%)	34 (91.9%)	1 (2.7%)	0 (0.0%)	4 (10.8%)	24 (56.8%)
Age group						
<30	20 (15.3%)	19 (95.0%)	0 (0.0%)	1 (5.0%)	4 (20.0%)	1 (5.0%)
30–60	86 (65.6%)	80 (93.0%)	0 (0.0%)	1 (1.2%)	7 (8.1%)	31 (36.0%)
>60	25 (18.3%)	23 (95.8%)	1 (2.7%)	0 (0.0%)	2 (8.3%)	14 (58.3%)

**Table 2:** Fungal culture results and distribution according to age and gender.

Patients	Fungus type			
	*Trichophyton rubrum *	*Candida *	*Trichophyton mentagrophytes *	All types of onychomycosis
	108 (82.3%)	19 (14.6%)	4 (3.1%)	131 (100.0%)
Gender				
Female	79 (84.0%)	12 (12.8%)	3 (3.2%)	94 (100.0%)
Male	29 (78.4%)	7 (18.9%)	1 (2.7%)	37 (100.0%)
Age Group				
<30	19 (95.0%)	1 (5.0%)	0 (0.0%)	20 (100.0%)
30–60	71 (82.6%)	12 (14.0%)	3 (3.5%)	86 (100.0%)
>60	17 (70.8%)	6 (25%)	1 (4.2%)	24 (100.0%)

**Table 3:** Onychomycosis severity index [15, 17] with age and gender relation.

Patients	Mild (1–5)	Moderate (6–15)	Severe (16–30)
	6 (4.6%)	37 (28.2%)	88 (67.2%)
Gender			
Female	4 (4.3%)	31 (33.0%)	59 (62.8%)
Male	2 (5.4%)	6 (16.2%)	29 (78.4%)
Age group			
<30	1 (5.0%)	5 (25.0%)	14 (70.0%)
30–60	5 (5.8%)	27 (31.4%)	54 (62.8%)
>60	0 (0.0%)	5 (20.8%)	19 (79.2%)

**Table 4:** Evaluation of patients to multiple nail involvements.

Patients	Great nail involvement	Multiple nail involvement
	74 (56.5%)	33 (25.2%)
Gender		
Female	58 (61.7%)	27 (28.7%)
Male	16 (43.2%)	6 (16.2%)
Age group		
<30 y.o	7 (35%)	5 (25.0%)
30–60	54 (62.8%)	23 (26.7%)
>60	12 (50%)	5 (20.8%)

**Table 5:** Laser treatment response according to age, gender, type of fungi, clinical type of onychomycosis, and location.

Patients	Excellent response (>75%)	Very good response (50–74)	Good response (25–49)	Moderate response (10–24%)	Low response (>9%)	No response (0%)
Gender						
Female	10 (10.6%)	44 (46.8%)	25 (26.6%)	10 (10.6%)	0 (0.0%)	5 (5.3%)
Male	2 (5.4%)	9 (24.3%)	16 (43.2%)	9 (24.3%)	0 (0.0%)	1 (2.7%)
Age						
<30 y.o	3 (15.0%)	4 (20.0%)	10 (50.0%)	2 (10.0%)	0 (0.0%)	1 (5.0%)
30–60	9 (10.5%)	37 (43.0%)	24 (27.9%)	13 (15.1%)	0 (0.0%)	3 (3.5%)
>60	0 (0.0%)	12 (50.0%)	6 (25.0%)	4 (16.7%)	0 (0.0%)	2 (8.3%)
Onychomycosis severity						
Mild	3 (50.0%)	2 (33.3%)	1 (16.7%)	0 (0.0%)	0 (0.0%)	0 (0.0%)
Moderate	5 (13.5%)	23 (62.2%)	9 (24.3%)	0 (0.0%)	0 (0.0%)	0 (0.0%)
Severe	4 (4.5%)	28 (31.8%)	31 (35.2%)	19 (21.6%)	0 (0.0%)	6 (6.8%)
Types of fungi						
* T. rubrum *	10 (9.3%)	51 (47.2%)	38 (35.2%)	8 (7.4%)	0 (0.0%)	1 (0.9%)
* Candida *	1 (5.3%)	1 (5.3%)	3 (15.8%)	10 (52.6%)	0 (0.0%)	4 (21.1%)
* Non dermatophytes *	1 (25.0%)	1 (25.0%)	0 (0.0%)	1	0 (0.0%)	1 (25.0%)
* T. mentographytes *	9 (9.4%)	48 (50.0%)	35 (36.5%)	4 (4.2%)	0 (0.0%)	0 (0.0%)
Clinical type of onychomycosis						
Distal subungual	9 (7.3%)	50 (40.7%)	40 (32.5%)	18 (14.6%)	0 (0.0%)	6 (4.9%)
Proximal subungual	0 (0.0%)	1 (100.0%)	0 (0.0%)	0 (0.0%)	0 (0.0%)	0 (0.0%)
Superficial white	1 (50.0%)	0 (0.0%)	0 (0.0%)	1 (50.0%)	0 (0.0%)	0 (0.0%)
Dystrophic	2 (4.3%)	14 (29.8%)	17 (36.2%)	11 (23.4%)	0 (0.0%)	3 (6.4%)
Lateral edge	2 (4.3%)	5 (38.5%)	5 (38.5%)	0 (0.0%)	0 (0.0%)	1 (7.7%)
Location						
Hand	0 (0.0%)	1 (9.1%)	3 (27.3%)	6 (54.5%)	0 (0.0%)	1 (9.1%)
Feet	9 (9.9%)	38 (41.8%)	27 (29.7%)	14 (15.4%)	0 (0.0%)	3 (3.3%)
